# “Outlaw” mutations in quasispecies of SARS-CoV-2 inhibit replication

**DOI:** 10.1080/22221751.2024.2368211

**Published:** 2024-06-25

**Authors:** Philippe Colson, Jacques Fantini, Jeremy Delerce, Wahiba Bader, Anthony Levasseur, Pierre Pontarotti, Christian Devaux, Didier Raoult

**Affiliations:** a IHU Méditerranée Infection, Marseille, France; b Microbes Evolution Phylogeny and Infections (MEPHI), Institut de Recherche pour le Développement (IRD), Aix-Marseille University, Marseille, France; c Assistance Publique-Hôpitaux de Marseille (AP-HM), Marseille, France; d INSERM UMR UA 16, Aix-Marseille Université, Marseille, France; e Department of Biological Sciences, Centre National de la Recherche 16 Scientifique (CNRS), Marseille, France

**Keywords:** SARS-CoV-2, genomics, mutations, quasispecies, evolution, next-generation sequencing

## Abstract

The evolution of SARS-CoV-2, the agent of COVID-19, has been remarkable for its high mutation potential, leading to the appearance of variants. Some mutations have never appeared in the published genomes, which represent consensus, or *bona fide* genomes. Here we tested the hypothesis that mutations that did not appear in consensus genomes were, in fact, as frequent as the mutations that appeared during the various epidemic episodes, but were not expressed because lethal. To identify these mutations, we analysed the genomes of 90 nasopharyngeal samples and the quasispecies determined by next-generation sequencing. Mutations observed in the quasispecies and not in the consensus genomes were considered to be lethal, what we called “outlaw” mutations. Among these mutations, we analysed the 21 most frequent. Eight of these “outlaws” were in the RNA polymerase and we were able to use a structural biology model and molecular dynamics simulations to demonstrate the functional incapacity of these mutated RNA polymerases. Three other mutations affected the spike, a major protein involved in the pathogenesis of COVID-19. Overall, by analysing the SARS-CoV-2 quasispecies obtained during sequencing, this method made it possible to identify “outlaws,” showing areas that could potentially become the target of treatments.

## Introduction

We have recently been able to identify “hyperfertile” mutations associated with the evolution of SARS-CoV-2 [1]. We have defined these mutations in this way because they are at the origin of phylogenetic nodes that have each generated more than 835 descendants. Other authors have described these mutations as belonging to a “whitelist” of mutations to indicate the benefit they confer to the virus [2]. All these studies were carried out on viral genome sequences, which consist of consensus genomes being selected and reproducing. These consensus, or *bona fide* genomes, which we call “democratic” genomes, are virtual molecules that are built with the most common nucleotides at each genome position, as determined during the assembly of sequence reads generated by next-generation sequencing (NGS). However, as particularly highlighted by the massive sequencing and analyses of HIV genes for genotypic antiviral drug resistance testing [3] and most recently by a global and unprecedent effort of genomic surveillance applied to SARS-CoV-2 [4], it is currently clear that viral quasispecies exist that are not necessarily encompassed in the “democratic” genomes.

For SARS-CoV-2, “hyperfertile” mutations appeared in viruses that circulated in Europe and are found at several notable positions [1]. The first amino acid substitution (P323L) is located in the NSP12 gene that encodes the RNA-dependent RNA polymerase (RdRp) and occurred in viruses that caused the pandemic onset after the Wuhan virus came into Europe [5]. It was reported to lead to a significant increase in the mutation rate and hence to an evolutivity that was probably critical in the development of a pandemic. In contrast, epidemics of SARS-CoV-1 and MERS-CoV did not exhibit such outcome with “hyperfertile” mutations, persistence of high levels of incidence for many months and over several years, and viral spread associated with new variants [6]. Regarding SARS-CoV-2, other key amino acid changes (firstly D614G) were located in the spike protein, which is explained by the predominant role of this protein in viral entry into host cells and hence in pathogenicity [7,8]. Paradoxically, several other favourable mutations were identified in so-called accessory genes, including ORF8, where they generated stop codons [9,10]. Thus, we described that these mutations in ORF8 obeyed the “Mistigri” rule as the loss of this gene led to a major rebound of virus incidence and spread, which in several cases was at the origin of a “hyperfertile” phylogenetic node [1]. In our present work, we wanted to identify the missing mutations that could be at the origin of abortive forms of the virus, which we called “outlaw” mutations. These mutations are referred to as belonging to a “blacklist” by other authors [2]. To achieve this, we used our database of SARS-CoV-2 genomes obtained by next-generation sequencing (NGS) and analysed the viral quasispecies in order to identify and locate the mutations that were the most common yet never expressed in the “democratic” genomes. Finally, we focused on frequent lethal mutations (not expressed in the “democratic” genomes) located in the RdRp in order to understand why these mutations could not be expressed.

## Materials and methods

### SARS-CoV-2 genomes

SARS-CoV-2 genomes from our centre are available from sequence databases including GenBank (https://www.ncbi.nlm.nih.gov/genbank/, [11]) and GISAID (https://gisaid.org/) [12], from the university hospital institute (IHU) Méditerranée Infection website at the following URL: https://www.mediterranee-infection.com/acces-ressources/donnees-pour-articles/60000-genomes/, and in Supplementary Table S1. As previously reported [1,10,13,14], these genomes had been obtained by NGS from respiratory samples collected from patients to diagnose SARS-CoV-2 infection and sent to our centre’s clinical diagnosis laboratory (IHU Méditerranée Infection, public and university hospitals of Marseille, southeastern France). In addition, the CoV-Spectrum web application (https://cov-spectrum.org/) [15] was used that allows determining mutations’ frequencies within SARS-CoV-2 genomes of the GISAID databases (https://gisaid.org/) [12].

### SARS-CoV-2 quasispecies analysis

The analysis of SARS-CoV-2 quasispecies was based on raw NGS data analysed in a previous study [13]. In short, this consisted of sequencing reads generated from 90 nasopharyngeal samples collected from SARS-CoV-2-positive patients between March and September 2020 through direct sequencing, in absence of prior PCR amplification that can affect sequencing accuracy, by the Illumina technology with the Nextera XT paired-end strategy on a MiSeq instrument (Illumina Inc., San Diego, CA, USA), as previously reported [14]. These reads had been mapped on the genome of the SARS-CoV-2 Wuhan-Hu-1 isolate (GenBank Accession no. NC_045512.2) using the CLC genomics workbench software v7 (https://digitalinsights.qiagen.com/) with thresholds of 0.8 for sequence coverage and 0.9 for nucleotide similarity. Only genomes with a mean depth of sequencing reads per position ≥50 and a coverage of the genome NC_045512.2 ≥ 90% were considered. Also, an intra-sample nucleotide diversity of 4% at a given nucleotide position was considered as the significant diversity threshold (as this corresponded to ≥2 reads per nucleotide position for a mean number of reads per position ≥50 at the genome scale). The 90 SARS-CoV-2 consensus genomes had been classified in lineages that circulated before or during the Alpha variant epidemic, based on the Pangolin (https://cov-lineages.org/resources/pangolin.html; [16]) and the Nextclade web applications (https://clades.nextstrain.org/; [17]). In 23, 9 and 5 cases, SARS-CoV-2 genomes were classified as belonging to 20A, 20B and 20C lineages, respectively, that derived from the Wuhan-Hu-1 isolate and circulated in our geographical area until May 2020. Additional SARS-CoV-2 genomes were classified as Pangolin lineages B.1.416 (or Marseille-1; *n* = 4 cases), B.1.177 (or Marseille-2; *n* = 8 cases), B.1 (or Marseille-3; *n* = 5 cases), B.1.160 (or Marseille-4; *n* = 13 cases), B.1.367 (or Marseille-5; *n* = 9 cases), B.1 (or Marseille-6; *n* = 2 cases), B.1.416.1 (or Marseille-7; *n* = 3 cases), B.1.1.269 (or Marseille-8; *n* = 3 cases), B.1.1.241 (or Marseille-9; *n* = 1 case), and B.1.221 (or Marseille-10; *n* = 5 cases). We sought for mutations in the SARS-CoV-2 quasispecies with the pileup function of the Pysam python module (https://www.python.org/) when analysing NGS reads, with parameters that include a quality score >13 as threshold. Complete genome mapping data generated by the CLC software and exported from the mapping output file as tab separated value files were analysed through an in-house Python (https://www.python.org/) script.

The frequencies of nucleotide mutations in consensus genomes from our centre were determined using an in-house Python script (https://www.python.org/) from the Nextclade output file (https://clades.nextstrain.org/; [17]). For other genomes, mutations’ frequencies within SARS-CoV-2 genomes were retrieved with the CoV-Spectrum online tool (https://cov-spectrum.org/) [15], which uses SARS-CoV-2 sequences obtained using any NGS technologies and procedures and stored in the GISAID databases (https://gisaid.org/) [12].

### “Outlaw” mutations

**Figure 1.**
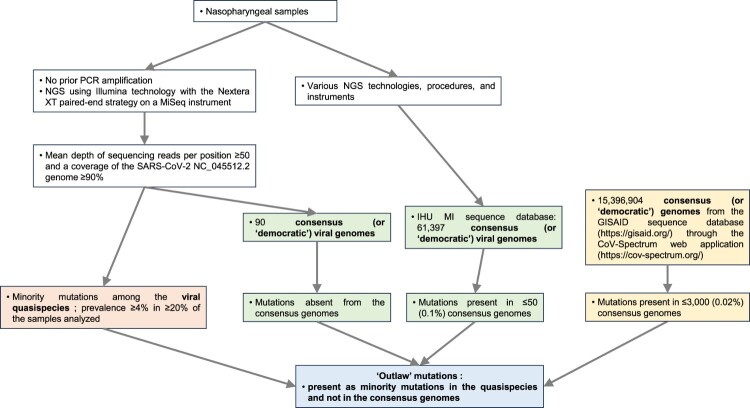
Schematic diagram to illustrate how SARS-CoV-2 genomes obtained by NGS were analysed to identify and locate the “outlaw” mutations, which are present in the virus quasipecies yet never expressed in the consensus, “democratic” genomes. Note: IHU MI, University Hospital Institute (IHU) Méditerranée Infection; NGS, next-generation sequencing.

We classified the mutations according to their frequencies among quasispecies as well as among consensus, or “democratic,” genomes. We gave the name “outlaw” to the mutations that were the most frequent among quasispecies, as defined by their presence with a prevalence of ≥4% in ≥20% of the genomes analysed [13], by their absence among “democratic” genomes from the 90 nasopharyngeal samples [1], and by their complete or almost complete absence among “democratic” genomes from our centre as defined by a presence in ≤50 (0.1%) of 61,397 genomes [1], and among “democratic” genomes from the GISAID database as assessed through the Cov-Spectrum web application (https://cov-spectrum.org/) [15] and defined by a presence in ≤3000 (0.02%) of 15,396,904 genomes (Figure 1).

Gene positions were taken from the UCSC genome browser web application (https://genome.ucsc.edu/cgi-bin/hgGateway) [18]. The four SARS-CoV-2 gene categories considered here had been defined in a previous study [1] and consisted of genes encoding (i) structural proteins; (ii) informational proteins (proteins involved in information storage and processing); (iii) other non-structural proteins; or (iv) accessory proteins.

### Protein structural analyses

The structural model of the SARS-CoV-2 RdRp (NSP12 gene product) was retrieved from pdb file 7bv2 [19]. An energy minimized model was generated with the Polak–Ribiere algorithm with the Bio-CHARMM force field in Hyperchem using a maximum of 3 × 10^5^ steps and a root mean square (RMS) gradient of 0.01 kcal/mol.Å as the convergence condition [20]. Mutations were introduced in RdRp with Deep View/Swiss-Pdb viewer, followed by several rounds of energy minimization as described previously [10]. Molecular dynamics simulations (MDS) were performed on a Dell workstation with the Hyperchem program (http://www.hypercubeusa.com) as previously described [21]. The systems were equilibrated at constant temperature (310 K) and constant pressure (1 atm) [20]. The energy of interaction of each RdRp-template complex was calculated with the ligand energy inspector function of Molegro Molecular Viewer (http://molexus.io/molegro-molecular-viewer), as described previously [22].

## Results

### Presence, prevalence and location of the “outlaw” mutations in the SARS-CoV-2 quasispecies

**Figure 2.**
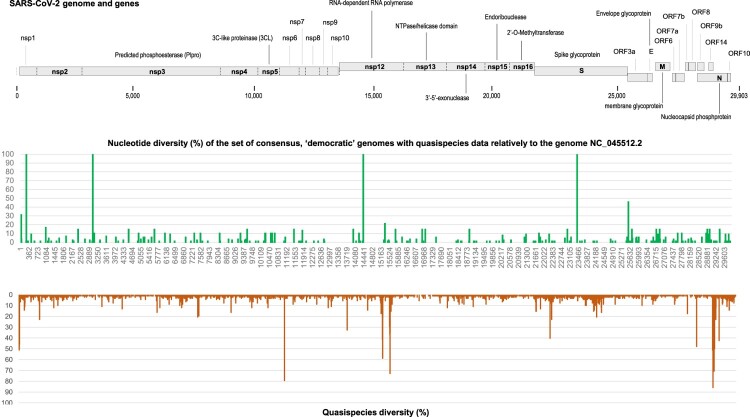
Quasispecies diversity along the viral genome in comparison with nucleotide diversity in consensus genomes for the same set of clinical samples. Notes: Quasispecies diversity is the proportion of samples who display a significant intra-sample nucleotide diversity. Nucleotide diversity is the proportion of sequencing reads that do not harbour the majority, consensus nucleotide.

Twenty-one positions in SARS-CoV-2 genomes analysed here were identified as harbouring “outlaw” mutations (Table 1; Figure 2) as they exhibited significant intra-sample nucleotide diversity but were exceptionally mutated in “democratic” genomes obtained from our centre and GISAID [15,16] relatively to the Wuhan-Hu-1 isolate genome. At these 21 positions, mean intra-sample nucleotide diversity ranged between 2.3% and 6.7%. In the 90 “democratic” genomes from quasispecies whose intra-sample nucleotide diversity was analysed, none of these positions were mutated. In the whole set of 61,397 “democratic” genomes obtained in our institute [1], the mean proportion of genomes harbouring these mutations was 0.003 ± 0.005% (0.000–0.020%). Finally, in a set of 15,396,904 “democratic” genomes analysed through the Cov-Spectrum online tool [16], this mean proportion was 0.007 ± 0.005% (0.001–0.017%).

The 21 nucleotide positions were located in NSP12 (the RdRp encoding gene) in 8 cases; in the Nucleocapsid encoding gene in 8 cases; in the Spike encoding gene in 3 cases; in NSP2, a non-structural gene that encodes a protein that may interact with other viral proteins, in one case; and in NSP3 that encodes a predicted phosphoesterase in one case [23] (Tables 1 and 2; Figure 2). None of these positions was located in accessory genes. We only focused hereafter on mutations located in the RdRp.

**Table 1.** Presence, location and prevalence of the “outlaw” mutations in the SARS-CoV-2 quasispecies.

Nucleotide position in the SARS-CoV-2 genome	SARS-CoV-2 gene	Number of consensus, “democratic” genomes, out of 90, that harbour the nucleotide mutation at a given position relatively to genome GenBank (https://www.ncbi.nlm.nih.gov/genbank/) Accession no. NC_045512.2	Mean intra-sample nucleotide diversity (%)	Maximum intra-sample nucleotide diversity (%)	Number of genomes harbouring the nucleotide mutation in the whole set of 61,397 “democratic” genomes obtained in our institute	Proportion (%) of genomes harbouring the nucleotide mutation in the whole set of 61,397 “democratic” genomes obtained in our institute	Number of genomes harbouring the nucleotide mutation in 15,396,904 “democratic” genomes from the GISAID (https://gisaid.org/) database according to the CoV-Spectrum web application (https://cov-spectrum.org/)	Proportion (%) of genomes harbouring the nucleotide mutation in 15,396,904 “democratic” genomes from the GISAID (https://gisaid.org/) database according to the CoV-Spectrum web application (https://cov-spectrum.org/)
868	NSP2	0	2.6	14.3	0	0.000	751	0.005
7459	NSP3	0	2.4	14.8	1	0.002	1813	0.012
13,693	NSP12	0	3.1	12.3	5	0.008	1030	0.007
15,157	NSP12	0	3.9	12.5	0	0.000	441	0.003
15,168	NSP12	0	4.8	9.6	1	0.002	2674	0.017
15,172	NSP12	0	2.3	6.9	1	0.002	255	0.002
15,455	NSP12	0	2.9	8.8	0	0.000	230	0.001
15,469	NSP12	0	3.5	10.0	0	0.000	204	0.001
15,474	NSP12	0	6.7	15.5	1	0.002	1081	0.007
15,479	NSP12	0	3.6	16.4	0	0.000	189	0.001
22,143	S	0	3.5	17.4	0	0.000	907	0.006
22,144	S	0	3.5	16.0	0	0.000	1133	0.007
24,089	S	0	2.3	33.3	5	0.008	1288	0.008
28,920	N	0	2.6	6.8	1	0.002	498	0.003
28,927	N	0	3.1	8.1	1	0.002	1521	0.010
28,931	N	0	4.1	12.7	12	0.020	2273	0.015
28,954	N	0	2.7	6.0	1	0.002	923	0.006
28,981	N	0	5.2	12.8	0	0.000	1374	0.009
29,049	N	0	2.7	10.1	1	0.002	904	0.006
29,187	N	0	3.8	16.7	0	0.000	573	0.004
29,188	N	0	4.1	16.7	7	0.011	2460	0.016

Note: S, spike encoding gene; N, nucleocapsid encoding gene; NSP12, RNA-dependent RNA polymerase encoding gene.

**Table 2.** Correspondence between mutated nucleotide positions in the NSP12 gene that encodes the RNA-dependent RNA polymerase gene and amino acid changes.

Nucleotide position relatively to SARS-CoV-2 genome GenBank (https://www.ncbi.nlm.nih.gov/genbank/) Accession no. NC_045512.2	SARS-CoV-2 gene	Nucleotide change	Codon change	Amino acid change
13693	NSP12	A13693U	ACA > UCA	T85S
15157	NSP12	C15157A	CAA > AAA	Q573K
15168	NSP12	G15168A	UUG > UUA	L576L
15172	NSP12	U15172A	UCA > ACA	S578T
15455	NSP12	C15455U	UCA > UUA	S672L
15469	NSP12	C15469A	CCA > ACA	P677T
15474	NSP12	U15474G	GGU > GGG	G678G
15479	NSP12	C15479A	ACC > AAC	T680N

### Structural analyses of “outlaw” mutations

#### Structural analysis of RdRp in complex with a template-primer RNA: effect of mutations generating an amino acid substitution

**Figure 3.**
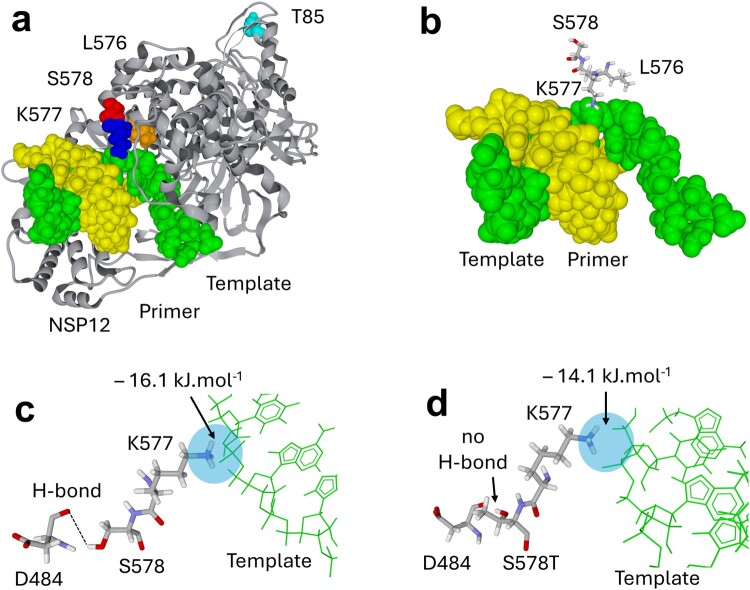
Localization of “outlaw” mutations in the structure of the SARS-CoV-2 RNA-dependent RNA polymerase (NSP12 gene product) in complex with a Template-Primer RNA. (a) Global view of RNA-dependent RNA polymerase (grey ribbons) with RNA template (green) and RNA primer (yellow). The positions of amino acid residues T85 (cyan), L576 (orange), K577 (blue) and S578 (red) are indicated. (b–c) Focus on the 576–578 triad near the template and D484. The arrows in panel c indicate a favourable interaction between the cationic group of K577 and the template. This interaction is optimized by a hydrogen bond between D484 and S578. (d) The S578T mutation breaks the hydrogen bond between amino acids 578 and 484, due to the methyl group of S578T. The energy of interaction of K577 with the template has been calculated for the wild-type (c) and the S578T mutant (d) after 100 ns of molecular dynamics simulation.

The structure of an energy-minimized model of RdRp in complex with a template-primer RNA is shown in Figure 3(a). Some of the “outlaw” mutations identified here are expected to affect the enzymatic activity of RdRp. Amino acid S578 does not directly interact with the template (Figure 3(b)). However, it is a critical residue that forms a stabilizing hydrogen bond with the peptidic NH group of D484 (Figure 3(c)). This ensures that the alpha-helix in position 563–581 comes in close contact with the long disordered loop in position 479–505. Mutation S578T is predicted to abolish the hydrogen bond with D484, leaving the 479–505 loop free to adopt alternative conformations that do not respect the functional 3D structure of the enzyme, potentially leading to a function loss. MDS of the S578T mutant confirmed the absence of hydrogen bond between S578T and D484 and the subsequent reorientation of the 479–505 loop. Indeed, the energy of interaction of D684 with residue 578 dropped from −8.3 kJ.mol^−1^ (hydrogen bond D484-S578, Figure 3(c)) to 0 kJ.mol^−1^ (no hydrogen bond between D484 and the S578T mutant, Figure 3(d)). This affected the energy of interaction of this residue with the template, ΔG being −16.1 kJ.mol^−1^ for the wild-type residue versus −14.1 kJ.mol^−1^ for the S578T mutant (hence, a 12.5% decrease). Overall, the energy of interaction decreased by 13.4% for the S578T mutant, ΔG being −233.7 kJ.mol^−1^, versus −269.5 kJ.mol^−1^ for the wild-type residue.

**Figure 4.**
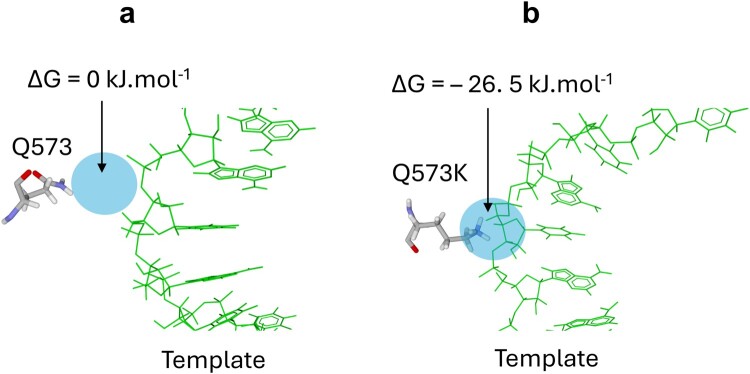
Effect of mutation Q573K in RNA-dependent RNA polymerase on the interaction with a Template-Primer RNA. (a) In the wild-type RNA-dependent RNA polymerase, Q573 is too far from the template to interact with it (Δ*G* = 0 kJ.mol^−1^). (b) In the case of Q573K, the cationic group of lysine interacts strongly with the template (Δ*G* = – 26. 5 kJ.mol^−1^), which may result in an inactive enzyme-substrate complex.

Other analysed mutations lie in the 572–573 region of RdRp. The amide group of the Q573 side chain is at 3.8 Å of the phosphate group linking template residues A11 and U12 (Figure 4(a)). This could, at best, allow the formation of a low energy hydrogen bond between the template and the enzyme (this possibility is not observed in the structure shown in Figure 4(a)). Mutation Q573K is expected to attract the template more strongly on the enzyme by replacing a loose hydrogen bond with strong electrostatic interactions. Such changes in the mode of enzyme–substrate interaction could functionally affect RdRp. Congruently, MDS suggested that Q573K considerably increased the energy of interaction between RdRp and the template. Starting from initial conditions (Figure 4(a)) with no interaction between the Q573 amide group and the template (a suspected weak hydrogen bond was not observed), Q573K resulted in a strong electrostatic interaction, with a ΔG of −26.5 kJ.mol^−1^ (Figure 4(b)). There is no other amino acid at such level of energy of interaction between wild-type enzyme and its substrates. Altogether these *in silico* data suggested that the complex between the Q573K mutant and the template might be too sticky to be fully functional.

## Discussion

In this work, we were able to implement a way of detecting unexpressed, also called “blacklist” mutations [2] or “outlaw” mutations, which are observed as minority mutations in the quasispecies and not in the consensus, “democratic” genomes. Interestingly, these “outlaw” mutations are found in the same genes as “hyperfertile” mutations [1], but at different nucleotide sites. In the case of the RdRp, where mutations are understandably not necessarily welcome, non-synonymous and synonymous mutations have been identified as “outlaw” mutations. In some cases that involved non-synonymous mutations, structural changes easily explain the loss of RdRp activity, which is not compatible with virus multiplication. Regarding synonymous mutations, structural explanation must be cautious. Nonetheless, these mutations may be associated with codons changes that modulate translation kinetics, which may alter protein folding in case it requires transitory stops of the ribosome induced by codons in the messenger RNA (mRNA) that match tRNA isoacceptors of low-abundance. For instance, it was thus reported in *Escherichia coli* that “silent” mutations inducing change to codons with highly abundant corresponding tRNA may affect the translational pausing, hence altering the co-translational folding of a bacterial protein (SufI) [24]. Another study conducted on *Escherichia coli* genes over 40,000 generations in a long-term evolution experiment indicated that purifying selection had a tendency to get rid of synonymous mutations that alter the secondary structures of mRNA, which implies these mutations might decrease bacterial fitness [25]. It was also reported that a synonymous mutation in the Multidrug Resistance 1 gene that encodes the P-glycoprotein corrupted the conformation of this protein in HeLa cells, and suggested that this mutation might alter the timing of co-translational folding and insertion of P-gp into the membrane, and consequently the protein function [26]. Besides, in hepatitis A virus, changes in optimization of codon usage in the capsid encoding region were found to tune translation kinetics for a proper folding of this protein [27], and in influenza A virus it was reported that synonymous mutations altering the structure of the nucleoprotein-encoding RNA could impact genome packaging and cause viral attenuation [28]. Altogether, these data indicate that synonymous mutations in genomes of RNA viruses may not be strictly neutral.

Overall, these observations are critical for the understanding of SARS-CoV-2 evolution. Some mutations have enabled spectacular and unprecedented development, while others were abortive. The frequency of “outlaw” mutations in quasispecies is surprising, suggesting a form of adaptive immunity with particular targeting of abortifacient viral genomic areas. We speculate that these areas could be the subject of hybridization with microRNA or other coding or non-coding elements of the human genome in response to viral aggression, possibly with the memory of past infection with viruses presenting sequences with a relative similarity, allowing recombination, which is only identifiable through the presence of such “lethal” mutations. This could correspond to an as yet undescribed mechanism of adaptive immunity. In fact, 128 human microRNAs were identified in the human lung epithelium as having the capability to target the SARS-CoV-2 genome [29].

Interestingly, a “outlaw” mutation may be the result of the action of cellular apolipoprotein B mRNA-editing enzyme catalytic polypeptide-like (APOBEC) enzymes, a described antiviral mechanism [30]. This APOBEC enzyme activity was also identified in the generation of “hyperfertile” mutations [1]. It is clear that this activity is random and not specific. Lastly, it is interesting to observe that ORF7 and ORF8 genes, which could harbour stop codons conferring no disadvantage to or being advantageous for the virus, and which were the subject of the greatest proportion of “hyperfertile,” “fertile,” and neutral or weakly deleterious mutations [1], were not at all the subject of “outlaw” mutations. This suggests that these genes probably only present disadvantages in the development of SARS-CoV-2 in humans. Thus, this seems in line with the general evolutionary strategy of coronaviruses, which are probably of animal origin, and which lose useless genes while adapting to humans, including ORF8 [10] that may have already disappeared from other human endemic coronaviruses [31]. In contrast, SARS-CoV and MERS coronavirus did not spread as prolifically as SARS-CoV-2, possibly because they lacked “hyperfertile” mutations in the RdRp and spike proteins and they kept the ORF8 “Mistigri” gene, although SARS-CoV experienced major deletions in that gene [32].

The present study has several potential limitations. First, we cannot exclude that NGS errors could contribute to the prevalence of some “outlaw” mutations, but error rate was previously estimated to be approximately 0.5% or lower with the Illumina technology on a MiSeq instrument [33–35]. Second, some “outlaw” mutations might be missed by NGS but with this Illumina technology, the estimated lowest detection threshold of minority variants was reported to be 0.5–1% and estimated sensitivity while detecting minor viral variants in a mixture of standards was reported to be 97.5% for a minor variant with a prevalence of 1% [36]. Beyond, other mutations than “outlaw” mutations can exist that are unseen through NGS in viral consensus, “democratic” genomes as well as in viral quasispecies, as they are too rare and/or too drastically deleterious for the virus. Third, structural explanation must be cautious regarding synonymous mutations as MDS is not valuable to be performed on the whole enzyme in such cases. Fourth, another limitation in the present study is the lack of further validation of the findings through *in vitro* experiments, which could be worthy to be done in future, dedicated works. Finally, sequential samples from patients chronically infected with SARS-CoV-2 were not investigated here, but this would be worthy to be performed in future studies to try observing the disappearance of “outlaw” mutations.

In conclusion, we believe that the deposition of all the quasispecies detected by SARS-CoV-2 genome NGS during the epidemic phases is essential to understand the evolution of the virus and to identify, among these quasispecies, those that will never be expressed in the *bona fide*, “democratic” genomes, making it possible to identify the most fragile genome areas and possibly, in the future, to prepare therapeutic tools based on the identification of these regions. Conserved viral genomic sites were previously deemed or demonstrated to be critical for virus replication and expansion [2,37]. Here, by searching deep in the viral quasispecies, we could identify the nucleotide and amino acid changes in viral gene sequences that lead to a fatal virus phenotype, and described that they can consist of both non-synonymous and synonymous mutations. Such “outlaw” mutations can be searched in already available NGS data or searched, and surveyed in future ones. Their identification may be contributive in the field of replication-defective live virus vaccines by conferring a fatal phenotype [38,39].

Beyond, the identification and characterization of “outlaw” mutations are worthy to help gaining a better insight into the structure–activity relationships in viral proteins and how synonymous mutations can have an impact on the generation and functionality of proteins. This might improve the targeting of particular sites in the viral proteins by monoclonal antibodies, or in the viral genes by small interfering RNA [40,41]. Anyway, here again, phenotypic studies should be performed at preliminary steps to confirm the detrimental effect of the mutations on the SARS-CoV-2 replication and propagation.

## Supplementary Material

Supplemental Material

## Data Availability

The set of SARS-CoV-2 genomes analysed in the present study are available from sequence databases including GenBank (https://www.ncbi.nlm.nih.gov/genbank/, [13]) and GISAID (https://gisaid.org/) [17], from the university hospital institute (IHU) Méditerranée Infection website at the following URL: https://www.mediterranee-infection.com/acces-ressources/donnees-pour-articles/60000-genomes/, and in Supplementary Table S1.
