# Supplementary material for: “Outlaw” mutations in quasispecies of SARS-CoV-2 inhibit replication
Source: Emerg Microbes Infect. 2024 Jun 25;13(1):2368211. doi: 10.1080/22221751.2024.2368211 (PMC11207925; doi:10.1080/22221751.2024.2368211)
Supplement: Supplemental Material [file TEMI_A_2368211_SM7437.docx]

**SUPPLEMENTARY MATERIAL**

**Supplementary Table S1. List of GenBank Accession no. for genome sequences of the present study**

ON266244

ON275096

ON275097

ON275107

ON275122

ON275143

ON275152

ON275162

ON275167

ON275190

ON275197

ON285612

ON285811

ON285812

ON285818

ON285970

ON286016

ON286037

ON286097

ON286124

ON286345

ON286354

ON286380

ON286404

ON286443

ON286466

ON286470

ON286481

ON297761

ON302767

ON302770

ON302771

ON302773

ON302779

ON302780

ON302792

ON302794

ON302796

ON302830

ON302844

ON302853

ON302862

ON302870

ON302878

ON302887

ON302891

ON302895

ON302897

ON302898

ON302899

ON302900

ON302901

ON302903

ON302908

ON302909

ON302911

ON302912

ON302925

ON302926

ON302928

ON302941

ON302944

ON302952

ON302955

ON302975

ON302987

ON302993

ON302996

ON302997

ON302998

ON303002

ON303003

ON303018

ON303022

ON303038

ON303048

ON303051

ON303052

ON303053

ON303054

ON303118

ON303132

ON303136

ON303156

ON303162

ON303168

ON303179

ON303211

ON303257

OP646528
